# A Genetic Variant in GPR126 Causing a Decreased Inclusion of Exon 6 Is Associated with Cartilage Development in Adolescent Idiopathic Scoliosis Population

**DOI:** 10.1155/2019/4678969

**Published:** 2019-02-11

**Authors:** Enjie Xu, Wei Shao, Heng Jiang, Tao Lin, Rui Gao, Xuhui Zhou

**Affiliations:** Department of Orthopedics, Changzheng Hospital, Second Military Medical University, Shanghai, China

## Abstract

Adolescent idiopathic scoliosis (AIS) is the most common spinal deformity disease in adolescents but its etiology and pathogenesis are still unclear. The current study aims to identify the relationship between single nucleotide polymorphisms (SNPs) of G protein-coupled receptor 126 (GPR126) gene and AIS predisposition. GPR126 contains 26 exons and alternative splicing of exon 6 and exon 25 produces 4 protein-coding transcripts. We genotyped SNPs of GPR126 gene around exon 6 and exon 25 in 131 Chinese AIS patients and 132 healthy controls and provided evidence that SNP rs41289839 G>A is strongly associated with AIS susceptibility. Linkage disequilibrium analysis suggests that rs41289839 and other AIS-related SNPs were in strong LD. Next, we demonstrated that rs41289839 G>A inhibits the inclusion of exon 6 during alternative splicing, resulting in a decreased expression level of exon 6-included transcript (GPR126-exon6^in^) relative to the exon 6 excluded transcript (GPR126-exon6^ex^) by minigene assay. Chondrogenic differentiation experiment showed that GPR126-exon6^in^ has a high expression level relative to GPR126-exon6^ex^ during chondrogenic differentiation of hMSCs. Our findings indicate that newly discovered SNP is related to cartilage development and may provide valuable insights into the etiology and pathogenesis of adolescent idiopathic scoliosis.

## 1. Introduction

Adolescent idiopathic scoliosis (AIS) is a structural, tridimensional spinal deformity with a Cobb angle greater than 10° [1, 2]. It occurs in 1–3% of the adolescent populations [3]. AIS has limited treatment options and is currently mainly used for brace or surgery. The etiology of AIS remains unknown, but genetic factors are considered to be the most important cause in many studies [4–6]. GWAS study in 1819 Japanese AIS cases and 25,939 controls by Kou et al. demonstrated that SNP rs6570507 of GPR126 is a new susceptibility locus for AIS [7]. Subsequent follow-up studies further confirmed the relationship between GPR126 and AIS [8–10]. The GPR126 protein is a member of the adhesion-G protein-coupled receptor (GPCR) family [11]. It is widely hypothesized that scoliosis is caused by abnormal skeletal growth [12]. A recent study reported that GPR126-null mice have limb posture abnormalities and growth failure [13]. GWAS studies suggest that human height is associated with SNPs in GPR126 in both children and adults [14–17]. In addition, an AIS related SNP rs6570507 was also associated with trunk length in European populations [15]. However, there is currently no research to explore the specific functions of the GPR126-SNP.

We further analyzed how GPR126-SNP regulates the gene function and even spinal development. The results of this research can be used for early diagnosis and development of new treatment methods for AIS.

## 2. Materials and Methods

### 2.1. Study Population

A total of 131 adolescent idiopathic scoliosis patients and 132 healthy controls in Shanghai Changzheng Hospital were enrolled in this study between October 2014 and February 2018. The standing posteroanterior radiographs were taken for each AIS patient. Cobb angle of the curves was measured, and the most severe curve was selected if more than one curve was discovered in one patient (Table 1). All patients in scoliosis group have at least one curve with a Cobb angle greater than 10 degrees. Blood samples were collected after obtaining informed consent from all participants or their parents. The study has been approved by the Ethical Committee of Shanghai Changzheng Hospital and conformed to the tenets of the Declaration of Helsinki.

### 2.2. PCR and Sequencing

GPR126 contains 26 exons and alternative splicing of exon 6 and exon 25 produces 4 protein-coding transcripts. We suspect that some SNPs in GPR126 may regulate the alternative splicing and finally alter the protein function of GPR126. Genomic DNA was extracted from peripheral blood of patients using the TIANamp Genomic DNA Kit (TIANGEN, China). The SNPs around exon 6 and exon 25 associated with splicing were analyzed. Sequencing primers: exon 6-F: 5′-TCTTTTGACAGACTCAGGAAACCA-3′; exon 6-R: 5′- AACTTGTTTCCTGCAGCAAATAAT-3′; exon 25-F: 5′- CTCAAACTCCTGGGCTCAAG -3′; exon 25-R: 5′- TCCTAGAAGGAGCCGCTTGC-3′. We also identified the genotype of rs6570507 by sequencing: primer: rs6570507-F: 5′-GAAAGATTTTCTGTGACATTCTC-3′; rs6570507-R: 5′-TGGTCAGGCTGGTCTCAA-3′. PCR conditions were set as follows: 1 min initial denaturation at 94°C, 30 cycles of 15 s denaturation at 94°C, 10 s annealing at 58°C, 1min extension at 72°C, and 10 min final extension at 72°C. The PCR products were analyzed using the sequencing system ABI3100 (Applied Biosystems).

### 2.3. Minigene Constructs

The exon trapping vector pCAS vector v1.0 (pcDNA3.1(-)/C1NH/SERPING1) was constructed as described by Gaildrat et al. [18]. We analyzed the sequence related to alternative splicing in intron 6 by RegRNA2.0 (http://regrna2.mbc.nctu.edu.tw/detection.html) and selected the 437 bp of 5′intron 6 and the 446 bp of 3′intron 6 for minigene assay. A 1053 bp genomic segment of GPR126 spanning exon 6, 437 bp of 5′intron 6, 446 bp of 3′intron 6, and exon 7 sequences was synthesized and then the fragments with the ancestral or mutant allele were cloned into pCAS vector v1.0 (Figure 2(a)). All constructs were sequenced to verify that contain the correct sequence.

### 2.4. Cell Culture and Transfection

DMEM medium contains 10% fetal bovine serum (FBS), penicillin (100 U/L), and streptomycin (100 mg/L). Human epithelial kidney 293 FT (HEK 293 FT) cells were cultured in DMEM medium and grow to approximately 70% to 80% confluence in a humidified atmosphere of 5% CO2 at 37°C. Cells were then transfected with 10 *μ*g plasmid DNA using OPTI-MEM medium and Lipofectamine 3000 (Invitrogen, USA) according to the manufacturer's instructions. Cells were harvested after 24 h transfection and total RNA was extracted with TRIzol (Invitrogen, USA).

### 2.5. Chondrogenic Differentiation

Chondrogenic differentiation of human mesenchymal stem cells (hMSCs) with rs41289839 genotype GG was applied by OriCell™ Human Mesenchymal Stem Cell Chondrogenic Differentiation Medium (Cyagen, USA). Chondrogenic medium contains high-glucose DMEM, 10 ng/mL recombinant human transforming growth factor-*β*3 (TGF-*β*3), 100 nM dexamethasone, 50 *μ*g/mL ascorbic acid, 1 mM sodium pyruvate, 40 *μ*g/mL proline, and ITS+ Supplement. MSCs were trypsinized, washed, and then resuspended in chondrogenic medium. 0.5 mL (2.5 × 10^5^ cells) of the cell suspension was transferred to a 15 mL polypropylene culture tube and centrifuged at 150 g for 5 minutes at room temperature. The caps of the tubes were loosened one half turn to allow gas exchange and the tubes were incubated in a humidified atmosphere of 5% CO2 at 37°C. The medium in each tube was completely replaced every 2-3 days. Chondrogenic pellets should be cultured for 3 to 21 days.

Pellets were fixed in formalin and embedded in paraffin for alcian blue stain. The slides were deparaffinized and hydrated in distilled water and stained for 30 minutes in alcian blue solution and washed in running tap water for 2 minutes and rinsed in distilled water. Images for analysis were captured and observed under light microscope. Blue staining indicated that hMSCS differentiated into chondrocytes and synthesized proteoglycans. Total RNA of pellet was extracted with TRIzol (Invitrogen, USA).

### 2.6. Reverse Transcription and PCR Analysis

A total of 1 *μ*g of RNA from the transfected cells and chondrogenic differentiation pellets were reverse transcribed into cDNA using oligo(dT)_18_ mRNA primer and SuperScript™ II Reverse Transcriptase in a 20 *μ*L reaction volume according to the manufacturer's instructions (Invitrogen, USA). The cDNA of transfected cells was amplified with primers: pCAS-F: 5′- GTCCGCTGACGTCGCC -3′; pCAS-R: 5′- GATCTGAGAGACTGAGGTGA-3′. The cDNA of pellets was amplified with primers: PLT-F: 5′- ACAGCTCTGCCTTGTTTGGA-3′; PLT-R: 5′- ACCTCAGGGTGACGAAGGAT-3′. PCR conditions were set as follows: an initial denaturation at 94°C for 1 min, 30 cycles at 94°C for 15 s, 58°C for 10 s, 72°C for 1 min, and a final extension at 72°C for 10 min. The PCR products were analyzed by electrophoresis on a 2% agarose gel. The signal ratio was obtained through comparing the band intensity of GPR126-exon6^in^ transcript with the band intensity of GPR126-exon6^ex^ transcript. The band intensity of each transcript was analyzed by Image-Pro Plus. We purified the PCR products of all bands using TIANgel Purification Kit (TIANGEN, China) and verified by Sanger sequencing.

### 2.7. Real-Time qPCR

The expression level of GPR126-exon6^in^ was determined by real-time qPCR (RT-qPCR) using SYBR-Premix Ex Taq (Takara, Japan) and ABI Prism 7900HT sequence detection system (Applied Biosystems, Carlsbad, CA). The genes were amplified using specific primers and human *β*-actin gene was used as an endogenous control. The PCR primer sequences used were as follows: exon 6 included GPR126-transcripts (GPR126-exon6^in^): forward: 5′- TACACCACCCACTGTCACCA-3′, reverse: 5′- ATTCTGCCACCTTGCTCTGT-3′; *β*-actin: forward: 5′- ACCGAGCGCGGCTACAG-3′, reverse: 5′- CTTAATGTCACGCACGATTTCC-3′. Data were analyzed using the comparative Ct method (2^-ΔΔCt^). Three separate experiments were performed for each group.

### 2.8. Statistical Analysis

Analysis of the data was performed using SPSS version 23.0, with p value < 0.05 considered statistically significant. The Student's* t*-test was used to compare the difference of GPR126-exon6^in^ expression between the ancestral type group and mutant type group. One-way analysis of variance (ANOVA) was used to analyze the differences between groups. The LSD method of multiple comparisons was used when the probability for ANOVA was statistically significant. We assessed the frequencies of the alleles and genotypes in patients and controls by *χ*^2^ test. Linkage disequilibrium analysis was carried out online using SHEsis (http://analysis.bio-x.cn) [19]. D' >0.7 and r^2^ >1/3 indicates strong LD between SNPs [20, 21].

## 3. Results

### 3.1. GPR126-SNP Identification and Linkage Disequilibrium Analysis

All individuals enrolled in our study were Chinese Han. Women in the case group account for a relatively high proportion (124/7), which is characteristic of AIS disease. The mean age of AIS patient was 13.94 ± 1.93 (range, 11-18). Since AIS occurs in adolescent with immature skeleton, the age of the controls was greater than 20 years to ensure they had mature bone (36.72 ± 16.84, range, 20-75). AIS patients were diagnosed at 12.91 ± 1.31 years old. The average cobb angle of the curve was 37.40°  ±  13.90 (range, 13-105°).

**Figure 1:**
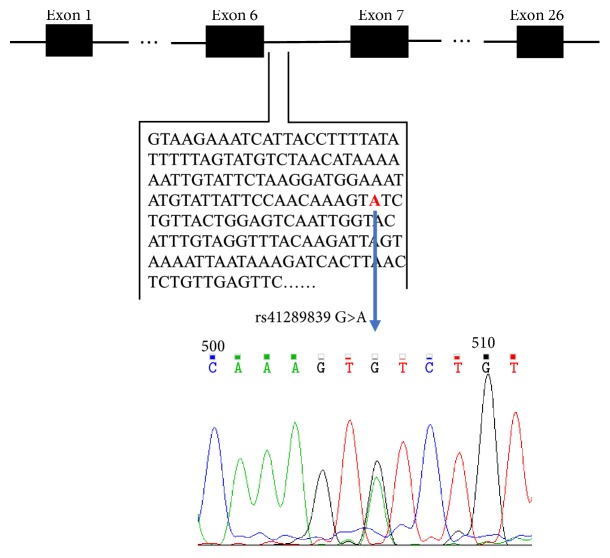
Identification of a novel SNP associated with AIS in GPR126.GPR126 gene consists of 26 exons and alternative splicing of exon 6 and exon 25 produces 4 protein-coding transcripts. A newly discovered SNP (rs41289839 G>A) at the junction of exon 6 and intron 6 was associated with AIS.

The GRP126 region was sequenced and compared to the control population. There were statistically significant differences in genotype and allele frequencies of rs41289839 (p = 1.55 × 10^−3^ and 2.68 × 10^−3^, respectively), indicating that we have newly discovered a SNP (rs41289839 G>A) at the junction of exon 6 and intron 6 associated with AIS (Table 2, Figure 1).

Rs6570507 was found to be associated with AIS in a large-scale genome-wide analysis [7] and this result was confirmed in our population (p = 4.05 ×10^−3^ and 4.02 × 10^−4^, respectively). Interestingly, linkage disequilibrium analysis by SHEsis showed that rs41289839 and rs6570507 were in strong LD (D' = 0.984, r^2^ = 0.461, Table 2).

### 3.2. The Newly Discovered SNP Regulates GPR126-Exon6 Splicing

Since intronic SNPs in the intron–exon boundaries have been reported to have effects on transcription factor binding sites and native splicing sites [22–24], we speculated that rs41289839 G>A may be associated with alternative splicing of exon 6. Splice prediction software HSF (http://www.umd.be/HSF3/) showed that the mutant type of this SNP may destroy the binding sites of the splice enhancement elements SPp55 and SC35 (Table 3).

**Figure 2:**
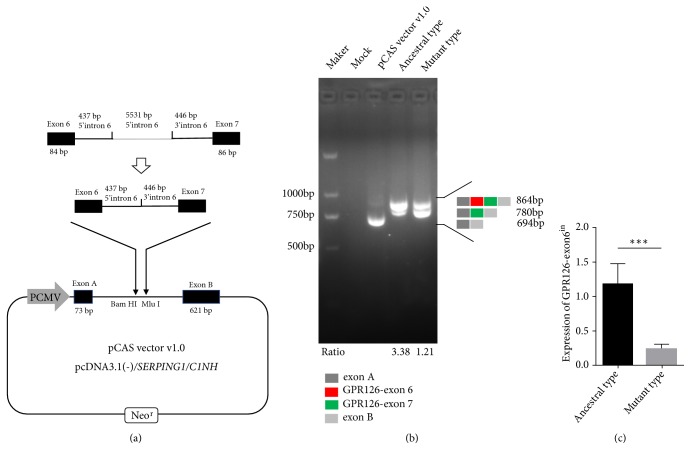
The exon trapping vector pCAS vector v1.0 used to assay SNP function. (a) The pCAS vector v1.0 contains 2 exons (exon A, exon B) and a functional intron. Ancestral or mutant plasmids containing exon 6, 437 bp of 5′intron 6, 446 bp of 3′intron 6 and exon7, and harboring either the G or A allele was separately cloned into the Bam HI and Mlu I clone site of the pCAS vector v1.0. Exon A: 73 bp; exon B: 621 bp; exon 6: 84 bp; exon7: 86 bp. (b) Agarose gel electrophoresis of RT-PCR products. Ancestral type: rs41289839-G; Mutant type: rs41289839-G; Lane 1: Marker; Lane 2: Mock; Lane 3: 694 bp (73bp+621bp); Lane 4: 864 bp (73 bp+84 bp+86 bp+621 bp), 780 bp (73 bp+86 bp+621 bp); Lane 5: 864 bp, 780 bp. There was a significant difference in the ratio of exon6^in^ to exon6^ex^ between Lane 4 and Lane 5. Ratio: exon6^in^/exon6^ex^. (c) The expression levels of GPR126-exon6^in^ in ancestral type group and mutant type group were detected by RT-qCPR.

Next, we constructed a minigene expression system and transfected it into 293FT cells (Figure 2(a)). The results of minigene assay demonstrated that rs41289839 G>A will inhibit the inclusion of exon 6 during alternative splicing, leading to a decreased expression level of exon 6-included transcript (GPR126-exon6^in^) relative to the exon6 excluded transcript (GPR126-exon6^ex^) (Figures 2(b) and 2(c)).

### 3.3. GPR126-Exon 6 Has an Important Role in Cartilage Development

**Figure 3:**
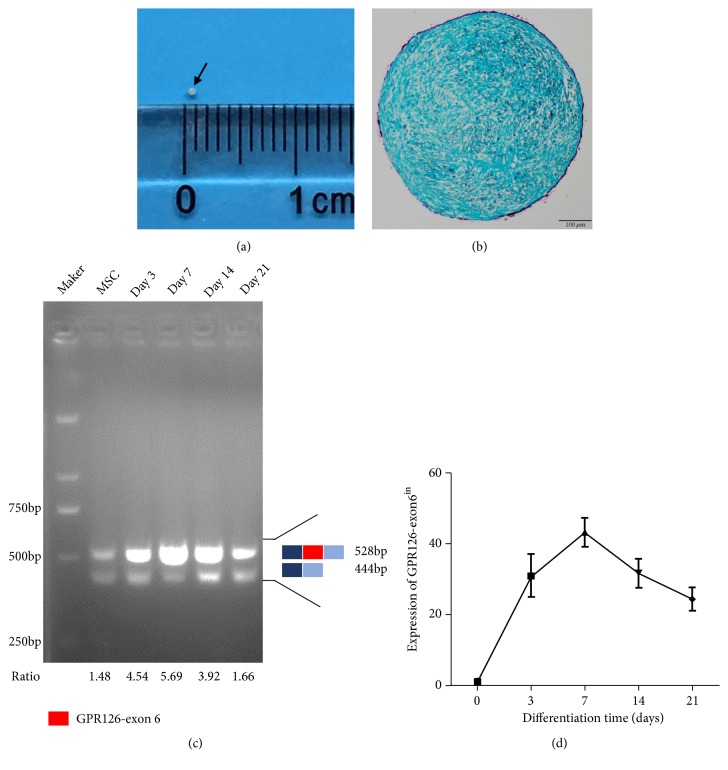
Inclusion of GPR126-exon6 during chondrogenic differentiation. (a) Chondrogenic differentiation of hMSCs for 21d. Black arrow: a chondrogenic pellet. (b) Alcian blue stain of chondrogenic pellet. (c) RT-PCR assay with primer PLT-F and PLT-R to monitor alternative splicing patterns of GPR126-exon6 during chondrogenic differentiation. Ratio: exon6^in^/exon6^ex^. (d) The expression levels of GPR126-exon6^in^ during chondrogenic differentiation were detected by RT-qCPR.

We performed an in vitro stem cell differentiation experiment to explore the role of exon 6 in cartilage development. Human mesenchymal stem cells were successfully differentiated into chondrocytes (Figures 3(a) and 3(b)). Reverse transcription PCR and real-time qPCR of chondrogenic pellets showed that GPR126-exon6^in^ has a high expression level relative to GPR126-exon6^ex^ during chondrogenic differentiation of hMSCs, suggesting that exon 6 may have important functions in cartilage development (Figures 3(c) and 3(d)). It indicated that a decreased inclusion of exon 6 caused by rs41289839 G>A may lead to cartilage malformation, which may be the cause of AIS.

## 4. Discussion

Alternative splicing is associated with multiple diseases such as spinal muscular atrophy [25], breast cancer [26], ovarian cancer [27], prostate cancer [28], and colon cancer [29]. Some studies have shown that alternative splicing is associated with intracellular localization and function of protein. The leptin gene can be translated into an isoform located in the nucleus and a cytoplasmic isoform, the former being a transcription factor and the latter having phosphatase activity [30, 31]. Typically the erythropoietin receptor is a membrane protein, but one of its splicing isoforms is a soluble protein [32]. The ability of proteins to bind to other proteins will change due to alternative splicing. An insulin splicing isoform (exon 11 skips) showed abnormally high affinity for IGF-II [33]. In addition, single nucleotide polymorphisms play an important role in exon splicing. CCND1 gene polymorphism (A870G) modulates alternative splicing and allows expression of an alternative cyclin D1 transcript (transcript cyclin D1b) which is correlated to the risk and/or severity of disease or drug response across a range of malignancies, including lung cancer [34]. A SNP in KLF6 intron generates a novel functional SRp40 DNA binding site and increases three alternatively spliced KLF6 isoforms [35] that are associated with prostate cancer [36] and lung cancer [37].

The etiology and pathogenesis of AIS are still uncertain [38]. Several familial surveys of idiopathic scoliosis provided strong evidence that genetic factors play a major role [39]. SNPs of ESR1 [40], ESR2 [41], MATN1 [42], MTNR1B [43], TPH1 [44], IL-6, and MMP-3 [45] have been reported to be related to AIS. Recently, a GWAS study found that GPR126 may also be a predisposing gene and rs6570507 of GPR126 was the most significantly associated SNP with AIS in Japanese [7]. In our study, we for the first time provided evidence for strong association of rs41289839 G>A with AIS susceptibility. Further functional analysis revealed that the newly discovered SNP regulates the splicing of GPR126-exon6.

AIS may be caused by abnormal cartilage development. Some researchers found that the imbalanced expression of sox9, collagen II, collagen X, and aggrecan on both sides of the spine may be the cause of scoliosis [46, 47]. Cartilage oligomeric matrix protein (COMP) played a role in maintaining the structural integrity of cartilage. Gerdhem found that COMP was lower expressed in idiopathic scoliosis children [48]. SHP2 plays important roles in cartilage development, and deletion of SHP2 in the cartilage causes scoliosis in mice [49]. On the other hand, some studies have shown that GPR126 plays a role in chondrocyte proliferation and cartilage formation. Sox9 is a chondrogenesis related factor, and studies have shown that the deletion of Sox9 leads to a decreased expression of GPR126 in the intervertebral disc of mice [50]. Later, some researchers found that GPR126 is highly expressed in cartilage of human and mice [7]. Deletion of GPR126 in mice cartilage caused “split” spine deformity [51]. In our study, we found that the expression level of GPR126-exon6^in^ transcript was higher than that of GPR126-exon6^ex^ transcript during chondrogenic differentiation of hMSCs, suggesting that exon 6 may have important functions in cartilage development. This result may explain how SNP affects cartilage development and finally causes scoliosis.

Linkage disequilibrium analysis in our study showed that there was a strong correlation between rs41289839 and rs6570507. In fact, the relationship between genetic marker and disease is complex. Some genetic markers correlate with the pathogenesis and pathology of the disease, and some have strong linkage disequilibrium with pathogenic sites [52]. We speculated that the newly discovered SNP is likely to be the pathogenic site of some AIS patients and rs6570507 is a “marker” for rs41289839.

This is a limited research because we did not detect GPR126 expression patterns in spine cartilage tissue of AIS patients. It is difficult to collect cartilage samples of AIS patients in posterior spinal orthopedic surgery. In the next work, we will collect spinal cartilage tissue in anterior spinal surgery with informed consent to define the expression pattern of GPR126 in AIS patients. Furthermore, there is currently no research explaining how GPR126 transcripts with/without exon 6 regulate cartilage development of spine. We will overexpress GPR126-exon6^in^ transcript or GPR126-exon6^ex^ transcript in hMSCs and apply chondrogenic differentiation to explore the function of exon 6 in cartilage development.

## 5. Conclusions

In conclusion, we examined the genetic association between GPR126 and AIS risk in Chinese populations, and the intronic SNP rs41289839 G>A was found to be significantly associated with AIS in Chinese populations. Further functional analysis revealed that the newly discovered SNP regulates alternative splicing of GPR126-exon6 and even cartilage development. These results could be helpful in understanding the etiology of AIS and developing drugs for AIS.

## Figures and Tables

**Table 1:** General data of patients and controls.

Variables	AIS cases	Controls
Ethnic group	Chinese Han	Chinese Han
Female/Male	124/7	62/70
Mean age ± SD (years)	13.94 ± 1.93	36.72 ± 16.84
Age range (years)	11-18	20-75
Age at diagnosis (years)	12.91 ± 1.31	NA
Mean Cobb angle ± SD (°)	37.40 ± 13.90	NA
Cobb angle range (°)	20-105	NA

**Table 2:** The allele and genotype frequencies of GPR126 gene polymorphisms in patients with AIS and controls.

SNP	group	Genotype frequency				Allele frequency			
		AA	AG	GG	p	A	G	OR(95%CI)	p
rs41289839	AIS	7 (0.053)	64 (0.49)	60 (0.46)	1.55 × 10^−3^	78 (0.30)	184 (0.70)	1.86 (1.24-2.80)	2.68 × 10^−3^
A/G	control	6 (0.045)	37 (0.28)	89 (0.67)		49 (0.19)	215 (0.81)		
rs6570507	AIS	36 (0.28)	53 (0.40)	42 (0.32)	4.05 × 10^−3^	125 (0.48)	137 (0.52)	1.89 (1.33-2.69)	4.02 × 10^−4^
A/G	control	17 (0.13)	52 (0.39)	63 (0.48)		86 (0.33)	178 (0.67)		

Linkage disequilibrium tests: D' = 0.984, r^2^ = 0.461

**Table 3:** Threshold scores of exonic splice enhancer (ESE) motifs associated with rs41289839 G>A.

Linked SR protein	Reference Motif (value 0-100)	Variation
SRp55	agtgtc (74.12)	Site broken -100
Sc35	gtctgtta (75.78)	Site broken -100

## Data Availability

The data used to support the findings of this study are available from the corresponding author upon request.

## Abbreviations

GPR126: G protein-coupled receptor 126
AIS: Adolescent idiopathic scoliosis
SNP: Single nucleotide polymorphism
LD: Linkage disequilibrium
hMSCs: Human mesenchymal stem cells
ESR1: Estrogen receptor 1
ESR2: Estrogen receptor 2
MATN1: Matrilin 1
MTNR1B: Melatonin receptor 1B
TPH1: Tryptophan hydroxylase 1
IL-6: Interleukin-6
MMP-3: Matrix metalloproteinase-3.
